# Notes and News

**Published:** 1896-02-01

**Authors:** 


### NOTES AND NEWS.

(COLLECTED AND COMMUNICATED.)

HIS ROYAL HIGHNESS THE DUKE OF YORK has consented to preside at the festival dinner of the Cabdrivers' Benevolent Association, to be held in the Whitehall Rooms, Hotel Metropole, on Wednesday, March 11th next.

AN American contemporary, referring to the New Year honours which have been conferred on English medical men, remarks: "None of them are known beyond the limits of the British Isles, except, perhaps, Sir Joseph Fayrer."

DR. JOHN S. BILLINGS, formerly of the United States Army, and late Professor of Hygiene in the University of Pennsylvania, has been appointed librarian of the New York Public Library. New York is to be congratulated on having secured the services of so eminent a man as Dr. Billings, whose experience renders him exceptionally fitted for such a post.

AMONGST the Christmas fare distributed to the London poor not the least interesting was the dinner given to 700 poor children in the Battersea Town Hall. This was provided by well-to-do children in South Australia, who sent this kindly recognition of brotherhood and token of goodwill all the way from Adelaide.

ANOTHER hospital worthy is about to give up his work and retire from the scene of his life's labours. Mr. F. Walker has been connected with St. Thomas's Hospital and has held the important office of steward to that institution for very many years. We have had occasion to confer and to correspond with Mr. Walker on many occasions during the last thirty years, and we have always found him most courteous and obliging, and possessed of a sound knowledge of everything which concerned the department of which he has had charge. We hope that Mr. Walker will receive an ample pension, and that he may be spared for many years to enjoy the restful ease to which he is justly entitled by his life's devotion to the best interests of St. Thomas's Hospital.

THE managers of J. R. Roberts' Stores have adopted an excellent custom, which might be followed with advantage by other firms which hold bazaars at Christmas. They charge one penny entrance fee to adults, the proceeds being sent to the West Ham Hospital. The amount presented this year amounted to £109, representing upwards of 26,000 entrances.

AN important case was settled recently in Dublin in which an injunction was granted to restrain a lessee from using a house as a private hospital. The covenant provided that the house should not be used to carry on an offensive trade. Although the hospital was for non-infectious cases, the Judges held that the agreement was infringed, and granted the injunction.

IT is the one aim and object of some people to find the most healthy spot to live in. The latest health resort we have heard extolled is Pentonville Prison, where during the past year, amongst 11,495 prisoners, there has only been one death. Whether the argument is most in favour of limited diet and plenty of work, or of the healthiness of the locality, or all three combined, it is not possible to state definitely.

THE objectionable condition of our railway carriages, especially those of the third class, is undeniable, and although complaint and dissatisfaction are rife, no steps are taken to improve matters. We do not usually look to Mexico for examples in sanitation, yet there the matter is receiving definite attention. A well-known medical man in Mexico is recommending that expectorating in the carriages should be prohibited under pain of severe penalties. The law would be difficult to enforce, but the community at large would be greatly benefited by so civilising a measure.

AT present there are very few general hospitals where any satisfactory arrangements exist for the reception of any but objects of charity; yet probably at every one of them well-to-do people intrude as trespassers in wards built for the use of the poor, squaring matters by what is really a douceur to the hospital for allowing this misappropriation of its beds. We would have it otherwise, and would make the reception of the well-to-do provide a source of revenue for the treatment of the poor; and we would do this without injuring the medical profession. No doubt some medical men will object to receive these fees. If so, let them hand them over to the Medical Benevolent Fund for the support of their sick and aged brethren. But we cannot doubt that many of the younger men will be very thankful to receive some sort of immediate return for their hospital work; something not quite so far off as that consulting practice which they all hope for; nor can we doubt that a small remuneration, by which young men could be helped to keep on their feet during those long years of waiting which nowadays tend to keep hospital appointments in the hands of a plutocracy of medicine, would be beneficial to the hospitals and to medical science. People think that the hospitals are officered by the cream of the schools. This might be so if the best men could be helped a little in their early struggles, but as it is few but those who are well off can afford to wait.

